# Retrograde urethrography, sonouretrography and magnetic resonance urethrography in evaluation of male urethral strictures. Should the novel methods become the new standard in radiological diagnosis of urethral stricture disease?

**DOI:** 10.1007/s11255-021-02994-5

**Published:** 2021-10-02

**Authors:** Frankiewicz Mikolaj, Markiet Karolina, Kozak Oliwia, Krukowski Jakub, Kałużny Adam, Belka Mariusz, Naumczyk Patrycja, Matuszewski Marcin

**Affiliations:** 1 Department of Urology, Faculty of Medicine, Medical University of Gdańsk, Gdańsk, Poland; 2 Department of Radiology, Faculty of Medicine, Medical University of Gdańsk, Gdańsk, Poland; 3 Department of Pharmaceutical Chemistry, Faculty of Pharmacy, Medical University of Gdańsk, Gdańsk, Poland; 4 Institute of Psychology, University of Gdansk, Gdańsk, Poland

**Keywords:** Urethral stricture, Magnetic resonance imaging, Conventional retrograde urethrography, Sonouretrography, Pelvic fracture

## Abstract

**Purpose:**

To verify which of the diagnostic modalities: Voiding cystouretrography (VCUG), Sonouretrography (SUG) or Magnetic resonance uretrography (MRU) is the most accurate in the assessment of urethral strictures in males and in what cases the application of novel imaging techniques benefits most.

**Methods:**

55 male patients with a diagnosis of urethral stricture, were enrolled in this prospective study. Initial diagnosis of urethral stricture was based on anamnesis, uroflowmetry and VCUG. Additional imaging procedures—SUG and MRU were performed before the surgery. Virtual models and 3D printed models of the urethra with the stricture were created based on the MRU data. Exact stricture length and location were evaluated by each radiological method and accuracy was verified intraoperatively. Agreement between SUG and MRU assessments of spongiofibrosis was evaluated. MRU images were independently interpreted by two radiologists (MRU 1, MRU 2) and rater reliability was calculated.

**Results:**

MRU was the most accurate [(95% CI 0.786–0.882), *p* < 0.0005] with an average overestimation of 1.145 mm (MRU 1) and 0.727 mm (MRU 2) as compared with the operative measure. VCUG was less accurate [(95% CI 0.536–0.769), *p* < 0.0005] with an average underestimation of 1.509 mm as compared with operative measure. SUG was the least accurate method [(95% CI 0.510–0.776), *p* < 0.0005] with an average overestimation of 2.127 mm as compared with the operative measure. There was almost perfect agreement of MRU interpretations between the radiologists.

**Conclusions:**

VCUG is still considered as a ‘gold standard’ in diagnosing urethral stricture disease despite its limitations. SUG and MRU provide extra guidance in preoperative planning and should be considered as supplemental for diagnosing urethral stricture. Combination of VCUG and SUG may be an optimal set of radiological tools for diagnosing patients with urethral strictures located in the penile urethra. MRU is the most accurate method and should particularly be considered in cases of post-traumatic or multiple strictures and strictures located in the posterior urethra.

## Introduction

Urethral stricture disease significantly impacts the patient’s quality of life. The incidence of urethral stricture (US) related to rapid development and accessibility to minimally invasive, transurethral urological procedures as well as urethral traumas linked to traffic or workplace accidents is increasing. The prevalence of US resulting from inflammation or traumatic catheter insertion seems to be stable [1].

Treatment results are not satisfactory. Medical centers lacking adequate experience often treat patients without sufficient preoperative evaluation, relying solely on anamnesis and uroflowmetry, sometimes complemented by urethroscopy. Due to accessibility, urethral dilatation and Direct Visual Internal Urethrotomy (DVIU) are still repeatedly performed, despite their high failure rates. On the contrary, precise preoperative diagnostics, along with the experience of the surgeon provide excellent outcomes even in complex cases [2].

An objective method of measuring voiding performance—uroflowmetry, supplemented by a subjective questionnaire that quantifies the severity of voiding symptoms—International Prostate Symptom Score (IPSS) remain the primary forms of evaluation of the patient with US. Yet, as recommended by an International Consultation on Urethral Strictures—uroflowmetry and IPSS should remain supplementary to imaging or cystourethroscopy in the initial diagnosis of US. Thus, despite numerous advantages, limited information provided by this routine prompted researchers to look for more accurate solutions [3–5].

For over a century, cystouretrography (CUG) and voiding cystouretrography (VCUG) have been the standard imaging procedures depicting location and length of the stricture. Novel methods such as sonouretrography (SUG) and magnetic resonance uretrography (MRU) gained recognition in recent years and are now established diagnostic tools in urethral stricture disease providing data regarding location and extension of the stricture, presence of spongiofibrosis and other periurethral conditions [6]. Additionally, MRU provides high resolution and excellent soft-tissue contrast allowing volumetric presentation of the results as a valuable supplement.

The above-mentioned diagnostic techniques applied preoperatively influence the optimal surgical approach and may often be a key element for successful treatment. Urethroplasty remains the ‘gold standard’ treatment with a high long-term success rate, yet besides adequate experience, requires precise preoperative qualification.

In our opinion, there is a need for complex evaluation of available imaging methods, to improve preoperative planning protocol in individual cases. Thus, the authors of this research, using their own clinical data, attempted to verify which of the diagnostic modalities: CUG/VCUG, SUG or MRU is the most accurate when compared to intraoperative findings, and in which patients an extension of diagnostics is most valuable.

## Methods

### Study group

Between September 2017 and October 2019, 55 male patients admitted to the Urology Department of our Institution with the diagnosis of urethral stricture disease, were enrolled in this single-center prospective study. The mean age was 57.9 (range 21–82). Diagnosis of US was based on clinical anamnesis, CUG/VCUG or urethroscopy and uroflowmetry performed prior to admission to the hospital or on the day of admission [7]. Medical interview covered information about the previous procedures within the urethra including DVIU, previous dilatations or any surgical treatment. Both newly diagnosed and patients with recurrent US, regardless of its cause or location, were included in the study. To objectively assess the accuracy of the studied imaging methods, each patient included in the study, had all imaging methods performed (CUG/VCUG, SUG and MRU) regardless of the cause and location of the US. In addition to the data on the imaging methods, the authors of this research provide supplementary data including results of uroflowmetry (*Q*_max_) and IPSS—both documented initially and 3–6 months after the surgery to assess the outcomes of the treatment. These data are available as an ‘Appendix’.

The study was approved by the Bioethics Committee in accordance with applicable requirements. Data presenting the location and etiology of the strictures in the studied group are shown in Table 1.

**Table 1:** Strictures location and etiology (*N* = 55 patients)

	Frequency	%
Stricture location		
Membranous	3	5.455
Membranous + prostatic	1	1.818
Bulbar	30	54.545
Bulbar + membranous	5	9.090
Penile	11	20
Penile + bulbar*	5	9.090
Etiology		
Iatrogenic		
Catheterization	9	16.363
TURP	19	34.545
URS	2	3.636
TURB	1	1.818
Prostatectomy	1	1.818
Idiopathic	5	9.090
Traumatic	17	30.909
Hypospadias + LS	1	1.818

*TURP* Transurethral resection of the prostate, *URS* Ureteroscopy, *TURB* Transurethral resection of bladder tumour, *LS* Lichen sclerosus

*In one case concomitant urethrocutaneous fistula was present

### Imaging techniques

#### CUG/VCUG

**Fig. 1:**
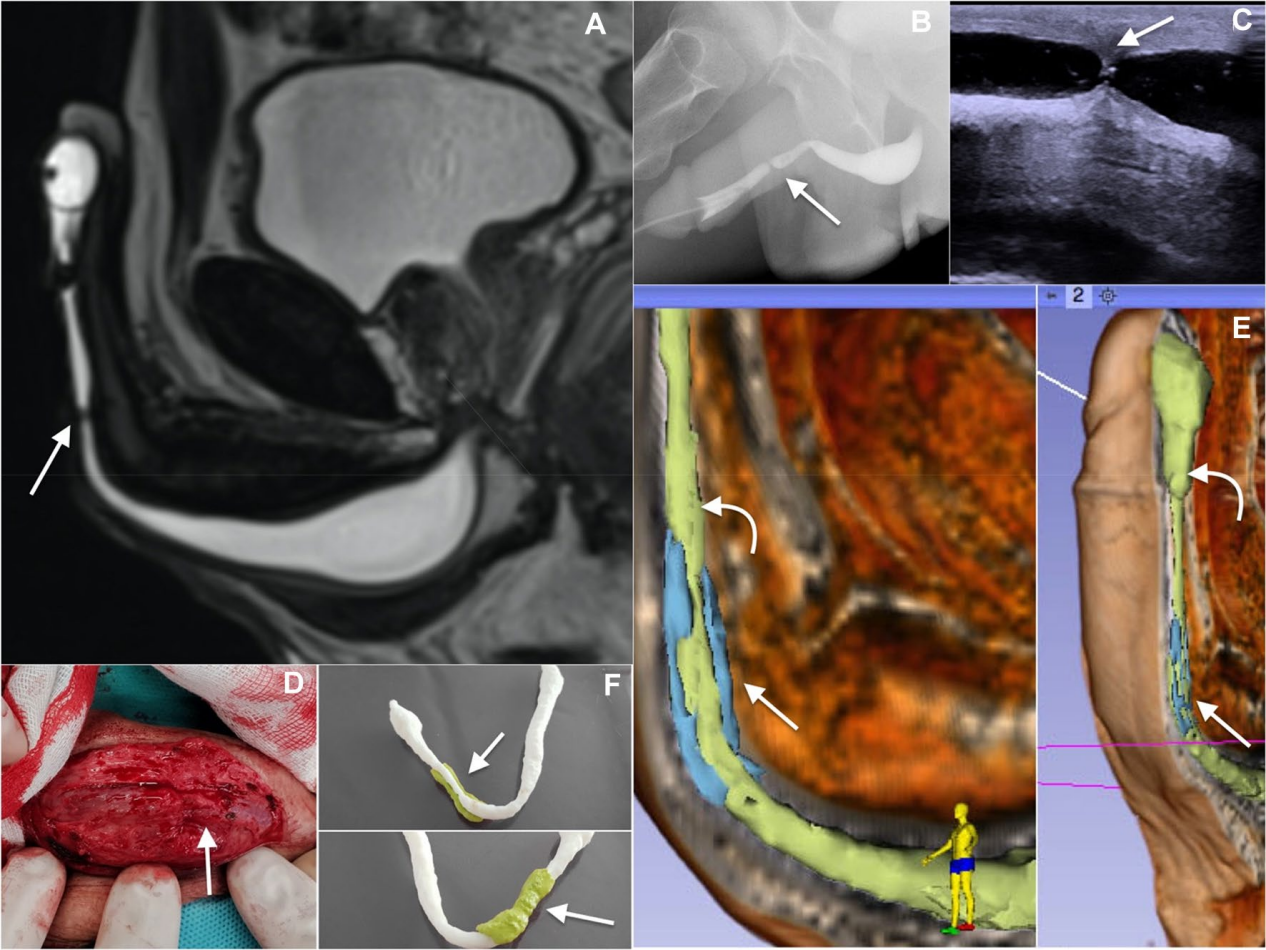
**A** MRU, sagittal T2-WI image of the urethra presenting a short stenosis of the penile part (arrow) with associated fibrosis (hipointense tissue modeling the lumen of the urethra at and around the level of stenosis). **B** VCUG, site of stenosis (arrow). **C** SUG, stenosis with associated fibrosis (arrow). **D** Surgery specimen, fibrotic tissue around the stricture (arrow). **E** Spongiofibrosis (arrow) surrounding the strictured urethra (curved arrow) segmented from the MRU Sagittal Contrast-enhanced T1-weighted sequence. **F** 3-D printout of the urethra based on raw data provided by MRU with visible site of stenosis (arrow), green color marks the extent of fibrotic changes

Both CUG and VCUG were performed according to a strictly defined scheme as described by McCallum and Colapinto [4]. The penis was placed in the lateral position, with the long axis of the penile urethra perpendicular to the femur. In cases of meatal stenosis or stricture in the penile urethra when introducing the catheter was not possible, a vascular cannula was used for the moment of contrast injection into the urethra. Images of maximum distension of the urethra were taken. In the second phase, VCUG was performed. The bladder was filled to the volume in which the patient reported urgency. Patients in an upright and half-side position were asked to start voiding and a series of images was taken (Fig. 1B, C). Each study was evaluated by a radiologist with over 10 years of experience in uroradiology.

#### SUG

BK Medical Flex Focus 800 ultrasound unit with a high-frequency linear transducer (18L5) was used. Frequencies varied from 6 to 18 MHz depending on the location of the stricture and amount of fat tissue in the perineal area. The technique of examination was as described by McAnninch et al. [8]. Stricture was detected at the site of urethra that did not comply with the stretching during saline infusion, contrary to a normal urethra that appears as a uniform, echo-free area, 8–10 mm in diameter. Alterations within the corpus spongiosum evaluated as hyperechoic areas compared to the hypoechoic healthy corpus spongiosum were classified as spongiofibrosis (Fig. 1C) [9, 10]. Each study was evaluated by a urologist with over 6 years of experience in urethral ultrasonography and over 200 urethral ultrasound examinations performed prior to this study.

#### MRU

MRU was performed in a 1.5 Tesla Unit (MagnetomAera, Siemens, Germany) with auto coil selection option with a 20-channel body and spine coil. After sterilization of the penis glans, sterile syringe filled with approximately 10–20 ml of anesthetic gel was introduced into the urethral meatus [11]. Gel was gently infused to distend the urethra. Foley’s catheter balloon was then filled up to 2 ml, and stabilized in fossa navicularis. With the patient in supine position, the penis was placed anteriorly and taped to the abdominal wall, allowing for optimal stretching of the urethra and fixing its position in the axis of midline (Fig. 1A).

Imaging protocol included sagittal T2-weighted Space seq. (TR 1600 TE 95 SL1,0i, FoV 301*301, matrix 288p*320), transverse T2-weighted TSE seq. (TR 5949 TE 97 SL3,0/0,45 FoV 356*356, matrix 359*448) and sagittal T1-weighted VIBE FS seq. (TR 3,4 TE 1,3 SL0.8i, FoV 310*371, matrix 229*352) prior and post i.v. contrast agent administration (delay time 3 and 10 min). Gadolinium contrast agent was used at standard dose of 0.1 mmol/kg of body weight (0.1 ml/kg of body weight) at a rate of 2–3 ml, followed by a 20-ml saline flush.

None of the patients had contraindications to magnetic resonance examination nor administration of contrast agent. Titanium implants/prostheses and previous pelvic orthopedic surgeries are, in most cases, not a contraindication to MRU [12]. Obtained images were independently evaluated by two radiologists (MRU 1, MRU 2) with 7 and 5-year experience in urogenital radiology relatively.

### MRU: virtual 3D model segmentation and 3D printing

Images obtained with MRU, were selected and transferred into 3D Slicer, a free open source software for medical image computing. Semi-manual segmentation of the urethra was conducted using the ‘Segment editor’ module. Sagittal T2-weighted Space sequence was used for urethral lumen segmentation and sagittal contrast-enhanced T1-weighted sequence for pathological spongiofibrotic tissue surrounding the strictured part of the urethra. The ‘Model maker’ module was then used to generate 3D virtual models of the urethra with the pathological surrounding tissue marked with a different colour (Fig. 1E). These selections were then exported to the standard tessellation language format (STL), widely used for 3D printing [13]. The Ultimaker3^™^ 3D-printer was used to create life-size, patient-specific 3D printed models of the urethra with the stricture. To separate the normal urethra from the surrounding pathological tissue as clearly as possible, dual extrusion multi-color printing was used (Fig. 1F).

### Radiological findings evaluation

Urethral stricture location, length and the extent of spongiofibrosis were evaluated. Other periurethral pathologies such as diverticula, tumours, fistulae and calcifications were also reported.

The accuracy of US measurement in CUG/VCUG, SUG and MRU was determined in each case in comparison to intraoperative measurement regarded as a reference value [14]. Mainly open surgery procedures—buccal mucosa graft (BMG) urethroplasty and excision with end-to-end urethroplasty—were applied. DVIU was considered and performed only as an initial procedure in short bulbar US.

To compare the agreement between SUG and MRU in the assessment of spongiofibrosis, McAninch and Chiou scales were used [8, 10]. These subjective classifications are routinely used for ultrasonographic evaluation of the degree of urethral damage and indirectly determinate the degree of spongiofibrosis [15]. For the purpose of this study, the authors decided to interpolate both classifications to MRU. CUG/VCUG are not included in this analysis as they do not provide information about spongiofibrosis.

Exact location of the stricture was evaluated by each radiological method and verified intraoperatively (Fig. 1D).

### Statistical analysis

#### Methodology

##### Urethral stricture length

The accuracy of US length measured by each radiological method (CUG/VCUG, SUG, MRU 1, MRU 2) was compared to the stricture length determined intraoperatively and calculated with weighted Cohen’s *κ*_w_ (as the ratings were non normally distributed).

**Fig. 2:**
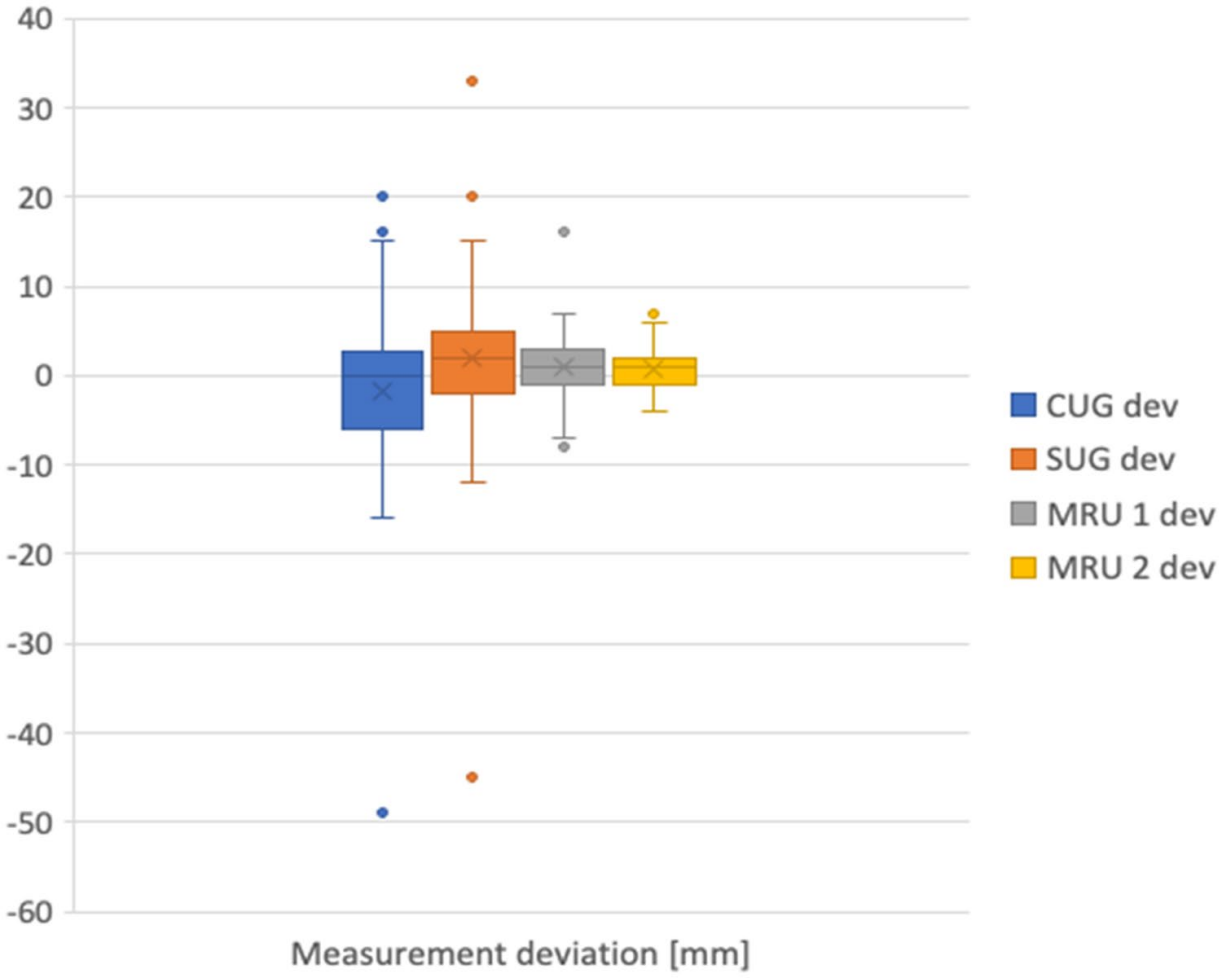
The deviation between the radiological measures and the operative measure (axis “0”). Negative scores represent underestimation, whereas positive scores—overestimation. Dots represent significant outliers in the measurements

Moreover, for each of them a deviation from the length determined during the operation was calculated (VCUG dev, SUG dev, MRU 1 dev, MRU 2 dev). Negative numbers indicated an underestimation of the measurement, whereas positive numbers—an overestimation with regards to intraoperative finding—Table 2 and Fig. 2.

**Table 2:** Length of the stricture—values measured by SUG, CUG, MRU 1 and MRU 2 and the operative measure

	Min	Max	Mean	SD
CUG	2	80	21.691	15.233
SUG	3	80	25.327	16.241
MRU 1	3	81	24.345	16.125
MRU 2	3	71	23.927	15.207
Operative measure	3	70	23.5	14.932
CUG dev	− 49	20	− 1.509	9.218
SUG dev	− 45	33	2.127	9.707
MRU 1 dev	− 8	16	1.145	3.67
MRU 2 dev	− 4	7	0.727	2.213

CUG/SUG/MRU measures given in [mm]

*SUG* Sonouretrography, *CUG* Cystouretrography, *MRU 1, 2* Magnetic resonance uretrography assessment of radiologist 1 and radiologist 2

To compare the radiological methods directly with each other, for each pair a Wilcoxon signed-rank test was performed to determine the significance of the difference between the measures—Table 3.

**Table 3:** Comparison of accuracy of SUG, CUG, MRU 1 and MRU 2 in measuring the length of the stricture (deviation from operative measure)

Comparison	*W*	*Z*	*p*	*r*
SUG vs CUG	301.5	− 3.098	0.002**	− 0.414
MRU 1 vs CUG	228	− 2.962	0.003**	− 0.396
MRU 2 vs CUG	415.5	− 2.323	0.02*	− 0.31
MRU 1 vs SUG	460	− 1.909	0.056	− 0.255
MRU 2 vs SUG	418	− 1.745	0.081	− 0.233
MRU 1 vs MRU 2	489	− 0.324	0.746	− 0.043

*SUG* Sonouretrography, *CUG* Cystouretrography, *MRU 1/2* Magnetic resonance uretrography assessment of radiologist 1 and radiologist 2, *W* Wilcoxon smallest sum of ranks, *Z* Wilcoxon signed-rank test, *p* significance, *r* effect size

*p < 0.05, **p < 0.01

In addition, as the MRU is not commonly evaluated by radiologists and might be considered demanding, the authors decided to verify the agreement between investigators. MRU images were independently evaluated by two radiologists (KM-MRU 1, OK-MRU 2). Inter-rater reliability of MRU assessment was calculated with weighted Cohen’s *κ*_w_ (as the ratings were non-normally distributed).

##### Spongiofibrosis

Spongiofibrosis was assessed based on Chiou [10] and McAninch [8] scales in SUG and adapted for MRU. The agreement between radiological measures (MRU vs SUG), as well as inter-rater reliability (MRU 1 vs MRU 2) was estimated with Cohen’s *κ*_w_ for both of the scales (Table 4). It must be emphasized, that this analysis tests the agreement between the SUG and MRI in the assessment of fibrosis, and not their accuracy. The only indisputable method of accuracy verification would be to perform histopathological evaluation.

**Table 4:** The agreement between spongiofibrosis estimation of SUG and MRU

McAnnich Scale				
	Kappa	95% CI		*p*
SUG vs MRU 1	0.8	0.72	0.881	0.0001
SUG vs MRU 2	0.57	0.471	0.669	0.0001
MRU 1 vs MRU 2	0.759	0.676	0.842	0.0001

Chiou Scale				
	Kappa	95% CI		*p*
SUG vs MRU 1	0.713	0.639	0.787	0.0001
SUG vs MRU 2	0.746	0.674	0.818	0.0001
MRU 1 vs MRU 2	0.479	0.392	0.565	0.0001

*SUG* Sonouretrography, *MRU 1/2* Magnetic resonance uretrography assessment of radiologist 1 and radiologist 2, *Kappa* Cohen’s kappa coefficient, *CI* Confidence Intervals

## Results

### Urethral stricture length

Accuracy of radiological methods compared to intraoperative measures are graphically presented on Fig. 2. Based on Landis and Koch [16] statistical methodology the agreement interpretations between radiological and operative measures varied from moderate to almost perfect:

- MRU was the most accurate [substantial to the almost perfect agreement, *κ*_w_ = 0.834 (95% CI 0.786–0.882), *p* < 0.0005] with average overestimation of 1.145 mm (MRU 1) and 0.727 mm (MRU 2) as compared with operative measure.
- CUG/VCUG was less accurate [moderate to a substantial agreement, *κ*_w_ = 0.653 (95% CI 0.536–0.769), *p* < 0.0005] with average underestimation of 1.509 mm as compared with operative measure.
- SUG was the least accurate method [*κ*_w_ = 0.643 (95% CI 0.510–0.776], *p* < 0.0005] with an average overestimation of 2.127 mm as compared with operative measure.

Yet, one must emphasize, that worse accuracy of SUG is a consequence of a few significant outliers in the SUG measurement, all of which were localized in the bulbar urethra. When verified with the Wilcoxon signed-rank test, the CUG/VCUG proved the most different measure (compared with MRU and SUG) with no significant differences between MRU and SUG.

### Spongiofibrosis assessment

Agreement between SUG and MRU assessments of spongiofibrosis using the McAnnich and Chiou scales was significant for all included assessments. Its degree varied from moderate to almost perfect—Table 4.

### Inter-rater reliability of MRU

There was almost perfect agreement between the radiologists’ assessment (MRU 1 vs MRU 2), *κ*_w_ = 0.883 (95% CI 0.838–0.928), *p* < 0.0005.

### Complications of VCUG, SUG and MRU procedures

The complication that occurred most frequently during imaging methods performed in this research, was local burning pain and discomfort and the site of stricture during the procedure. During VCUG, it was reported by 31 (56.4%) patients, during SUG—22 (40%) patients and 18 (32.7%) patients during MRU. Contrast extravasation was reported in 2 (3.63%) patients during VCUG. No adverse systemic reactions occurred in these patients. No serious bleeding that required intervention was observed in any of the studies. Infections in the postoperative period as a complication of imaging examinations were not reported by the authors, as the short time from examination to surgery prevented a reliable evaluation.

## Discussion

Improvement of US treatment results is of the utmost importance in reference to the gradual increase of the number of patients with iatrogenic stenosis. Non-invasive diagnostic methods, such as uroflowmetry and voiding symptom assessment questionnaire—IPSS, despite being highly useful, do not provide enough data for a clear diagnosis, and are, therefore, considered as supplementary tools.

To understand the essence of preoperative imaging, the pathophysiological mechanism of US formation should be explored, as the stricture is not limited to intraluminal pathology. The epithelial layer at the site of a stricture is much thicker than in healthy urethra due to changes within collagen fibers and elastin bundles densely packed around altered urethra [17]. This explains poor outcomes of repeated DVIU, resulting in scarring within the corpus spongiosum leading to spongiofibrosis.

Therefore, the role of imaging tool that would expose not only the exact length of the stricture, but also the extent of fibrosis, is undeniable. It is also confirmed by the treatment results—in patients with extensive spongiofibrosis the best are achieved by excision of the fibrotic fragment, followed by an end-to-end anastomosis of the two healthy ends. Yet, if the extent of fibrosis is less marked, urethroplasty with BMG may be the method of choice [18].

Undoubtedly, CUG/VCUG remains particularly valuable for initial diagnosis and planning of urethral reconstruction. Available, simple and repetitive, with satisfactory accuracy of diagnosing location and length of the stricture, as shown by this study, should still be the first choice. The value of urethroscopy cannot be underestimated either. However, with the painful and/or often impossible introduction of the tool in cases of significant or complete stricture, its application is limited and thus was not subjected to detailed evaluation in this paper.

As demonstrated, CUG/VCUG reveals tendency to underestimate the stricture length. This may be explained by its inability to assess the pathological periurethral tissue. To overcome this limitation, a combination of CUG/VCUG and SUG may be an optimal set of radiological tools for diagnosing patients with US, especially located in the penile urethra. It must be underlined that some significant outliers in the SUG measurement occurred in the studied group as mentioned previously. All of these errors were localized in the bulbar or membranous urethra, none in the penile urethra. This fully reflects the technical difficulties associated with performing SUG in the bulbar urethra and being almost impossible in the posterior urethra, despite proper patient positioning and high operator experience [19]. Transrectal sonographic approach may significantly improve the evaluation of posterior urethra, however, data on this technique and its accuracy is still limited [20]. Moreover, SUG is a subjective, operator-dependent procedure. Thus, despite its advantages, it may result in significant misdiagnosis of US, if excessive pressure of the probe is exerted.

Limitations in imaging of the posterior urethra were also reported by other authors in relation to the MRU examination. Sung et al. [11] assumed that proximal parts of the posterior urethra are rarely shown on MRU. However, based on our results, technical limitations of the MRU may be overcome by a strictly defined protocol of patient preparation with emphasis on patient positioning and accurate urethra distension. As such, MRU provides not only information about the length if the stricture, but also on surrounding tissues and pelvic anatomy, crucial in treatment planning.

Taking into account the accuracy of the radiological methods tested in this study as compared to the operative findings, MRU with average overestimation of 1.145 mm (MRU 1) and 0.727 mm (MRU 2) is the most accurate examination. Moreover, the almost perfect agreement between the radiologists’ assessment (MRU 1 vs MRU 2) indicates that MRU allows an objective assessment of the pathology regardless of an experienced investigator.

**Fig. 3:**
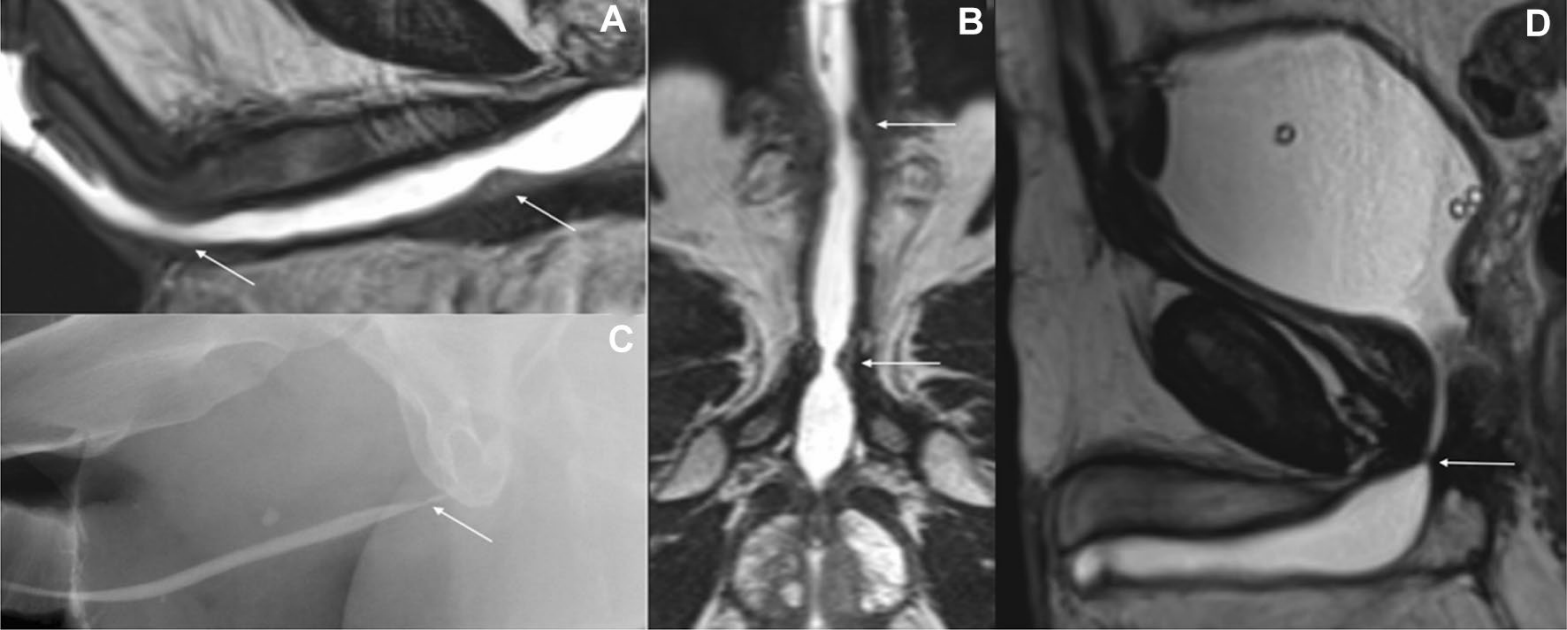
MRU sagittal T2-WI image (**A**) and reformat through the lumen of the urethra (**B**) present two stenoses within the bulbar part and at the border of the bulbar and penile part with surrounding fibrosis (arrows). **C** VCUG. Stenosis in the bulbar part of the urethra is also visualized in urethrography. **D** MRU sagittal T2-weighted image presents a short stenosis (arrow) in the bulbar part of the urethra, impossible to demonstrate in SUG

Yet, considering the higher cost and lower availability of MRU as compared with CUG/VCUG and SUG, its benefits will be the most valuable particularly in cases of post-traumatic US, multiple strictures (Fig. 3A, B) and strictures of the posterior urethra (Fig. 3D). MRU is also of particular value in patients after blunt force pelvic fracture who suffer from pelvic fracture urethral injury (PFUI) as both the direction and degree of prostatic displacement may be clearly depicted [21].

Moreover, 3D reconstructions and printed models allow the presentation of the pathology in an accessible form despite its complex nature. This noticeably valuable factor may be implemented in educating surgeons inexperienced in reconstructive urology. The ability to create such accurate models based on real urethral pathologies may also play a part in ongoing researches exploring tissue engineering in urethral reconstruction [22].

It is worth noting that CUG/VCUG, although less accurate in measuring the stricture length—with an average underestimation of 1.509 mm as compared with operative measure, also provides high accuracy, being less expensive and widely available. The disadvantage, however, is the lack of assessment of the pathology around the urethra and more frequent complaints reported by patients during intraurethral administration of the contrast.

In our study, SUG was the least accurate method with average overestimation of 2.127 mm as compared with the operative measure. Yet, in further analysis of the data—worse accuracy of SUG is a consequence of significant errors in measurements of strictures localized in the proximal and bulbar urethra, while in strictures in the distal and penile parts, no significant differences between MRU and SUG were revealed. Thus, should be routinely be considered in strictures located in the distal urethra and especially in the penile urethra being easily accessible to the examiner.

The authors of this study are aware of its limitations. First, this is a single-center study with a moderate number of patients. Second, intraoperative visual evaluation, despite being used by other authors, is not an ideal reference method for the assessment of spongiofibrosis [23]. Histopathological analysis of the excised urethra would be the most accurate reference, however, obtaining reliable specimens is a significant obstacle. The number of urethral specimens analyzed in this study prevented a statistically significant evaluation. Thus, a large multi-center clinical study taking into account the above limitations would undeniably let physicians gain valuable insights into urethral stricture disease.

## Conclusions

Due to cost-effectiveness, availability and satisfactory accuracy, VCUG is still considered as a ‘gold standard’ in diagnosing urethral stricture disease despite its limitations.

Evaluation of the extent of spongiofibrosis should become an integral part of urethral stricture disease diagnostics as well as the length and location of strictures.

SUG and MRU should be taken into account in all cases when the diagnosis or the choice of optimal surgical method is uncertain. Combination of CUG/VCUG and SUG may be an optimal set of radiological tools for diagnosing patients with urethral strictures, especially located in the penile urethra.

MRU should be implemented particularly in cases of post-traumatic urethral strictures, multiple strictures and strictures located in the posterior urethra. MRU may also be successfully implemented in urethral reconstruction training centers due to the educational value of volumetric reconstructions and 3D printed models.

## Data Availability

Not applicable.

## Appendix

**Table:**

	Age	Etiology	Stricture location (urethra)	Previous procedures	Surgery	Stricture length [mm]						Fibrosis (McAninch)				Fibrosis (Chiou)				IPSS		*Q*_max_	
						CUG/VCUG	SUG	MRU1	MRU2	Intraoperative	Histopathology	SUG	MRU 1	MRU 2	Histopathology	SUG	MRU 1	MRU 2	Histpathology	At admission	> 3 m after operation	At admission	> 3 m after operation
1	41	Trauma (PFUI)	Bulbar	Newly diagnosed	End-to-end	10	12	13	14	15	14	S	S	S	S	3	3	3	4	35	6	0	18.5
2	53	Traumatic catheterisation	Bulbar	Newly diagnosed	End-to-end	18	21	18	19	18	19	S	S	MD	S	3	2	2	3	14	1	5.9	23
3	69	Post TURP	Bulbar	Newly diagnosed	End-to-end	6	14	12	7	6	12	S	S	S	MD	3	3	3	1	23	3	8.1	15.6
4	41	Trauma (PFUI)	Bulbar	Newly diagnosed	End-to-end	12	25	16	17	15	15	S	S	S	S	3	3	3	3	32	4	3.3	30.3
5	21	Hypospadias and LS	Penile	Newly diagnosed	BMG	18	33	29	27	25	–	S	S	S	–	5	5	5	–	33	7	0	10
6	37	Traumatic catheterisation	Penile	Newly diagnosed	BMG	27	42	32	38	37	–	S	MD	MD	–	5	4	4	–	31	9	5	24
7	34	Traumatic catheterisation	Bulbar	2 × DVIU	End-to-end	17	30	19	19	17	–	S	S	MD	–	4	2	2	–	28	2	0.5	45.5
8	57	Post TURP	Penile	Newly diagnosed	BMG	29	36	32	38	40	–	S	S	S	–	5	5	5	–	16	1	7.3	22
9	63	Traumatic catheterisation	Bulbar	10 × DVIU	BMG	29	28	31	31	30	–	M	M	M	–	4	4	4	–	23	20	17.6	17.9
10	33	Idiopathic	Bulbar	6 × DVIU	BMG	25	25	25	24	28	–	S	S	S	–	3	3	3	–	17	16	7.7	9
11	61	Idiopathic	Bulbar	18 × DVIU	End-to-end	19	22	27	23	25	20	S	S	S	S	5	5	3	3	26	17	0	4
12	67	Traumatic catheterisation	Penile	Newly diagnosed	Orandi flap	21	30	29	28	30	–	S	S	S	–	5	5	5	–	25	30	0.5	8.3
13	51	Trauma (penile fracture)	Penile	Newly diagnosed	BMG	5	20	3	3	5	–	S	S	S	–	4	3	3	–	32	4	7.7	28.7
14	67	Post TURP	Bulbar	DVIU	End-to-end	12	33	23	21	19	20	S	S	S	S	3	3	3	3	23	11	4.3	20.7
15	54	Trauma (PFUI)	Bulbar	1 × DVIU	Not performed	NS	NS	NS	NS	–	–	–	–	–	–	–	–	–	–	35	30	12	12.5
16	82	Post TURP	Bulbar	4 × DVIU	End-to-end	36	55	38	34	35	32	S	S	MD	S	5	5	5	5	33	4	3	18
17	72	Post TURP	Penile	1 × DVIU	BMG	14	25	23	28	30	–	MD	MD	MD	–	3	3	4	–	32	19	5.1	24
18	87	Traumatic catheterisation	Bulbar	2 × DVIU	End-to-end	10	21	15	10	12	–	S	MD	MD	–	3	2	2	–	27	20	11	12
19	64	Idiopathic	Bulbar	1 × DVIU	End-to-end	10	16	11	11	10	–	S	S	S	–	3	2	2	–	13	3	16.5	21
20	62	Trauma (straddle injury)	Bulbar and membranous	1 × DVIU	End-to-end	10	12	11	12	15	12	S	MD	MD	S	3	2	2	2	12	7	0	16
21	67	Post TURP	Penile and bulbar	5 × DVIU	BMG	9	22	9	10	10	–	MD	M	M	–	2	2	2	–	30	1	8.5	10.3
22	51	Trauma (straddle injury)	Bulbar	2 × DVIU	End-to-end	28	23	34	37	35	–	S	S	S	–	5	5	5	–	16	4	0	10
23	74	Traumatic catheterisation	Bulbar	3 × DVIU	BMG	12	27	20	21	20	–	MD	MD	MD	–	2	2	2	–	29	4	5	17
24	39	Idiopathic	Bulbar	5 × DVIU	BMG	18	30	18	18	18	–	MD	MD	MD	–	2	2	2	–	21	7	9	28.7
25	71	Trauma (PFUI)	Bulbar	13 × DVIU	End-to-end	25	20	28	27	25	–	S	S	MD	–	5	5	5	–	21	9	6	12
26	46	Trauma (PFUI) + TURP	Bulbar	4 × DVIU	Cystostomy	65	27	65	66	–	–	S	S	S	–	5	5	5	–	22	35	0	–
27	56	Trauma (PFUI)	Bulbar and membranous	1 × UP	End-to-end	21	20	21	20.5	20	–	S	S	S	–	3	3	3	–	26	2	11	16
28	54	Trauma (PFUI)	Bulbar	2 × UP	DVIU	4	5	5	5	4	–	S	S	S	–	2	2	2	–	35	4	17	21
29	68	RT	Bulbar	1 × DVIU	BMG	25	25	25	25	25	–	S	S	S	–	3	3	5	–	31	30	4	15.1
30	38	Trauma (straddle injury)	Penile and bulbar	3 × DVIU + circumcision	BMG	80	80	81	65	65	–	MD	MD	MD	–	5	5	4	–	22	7	11.9	25.6
31	34	Trauma (PFUI)	Bulbar and membranous	3 × DVIU	End-to-end	11	12	12	14	15	–	S	S	S	–	3	3	3	–	16	9	0	55
32	73	Post prostatectomy + RT + AMS	Bulbar	AMS	End-to-end	17	18	17	19.5	18	–	S	S	S	–	3	3	3	–	23	35	0	9
33	65	Post URSL	Bulbar and membranous	4 × DVIU	BMG	30	22	24	24	25	–	MD	MD	MD	–	3	3	3	–	32	1	9.1	18.2
34	63	Post TURP	Bulbar	2 × DVIU	BMG	39	54	47	48	45	–	S	S	MD	–	5	5	3	–	16	11	4.4	21
35	43	Traumatic catheterisation	Bulbar	2 × DVIU	BMG	30	28	30	33	30	–	S	S	S	–	5	5	5	–	30	10	11.7	35.1
36	75	Post TURP	Membranous and prostatic	DVIU + UP	DVIU	15	12	10	10	10	–	S	S	S	–	3	3	3	–	23	3	4.1	14
37	68	Post TURP	Membranous	2 × DVIU + TURP	End-to-end	4	7	5	5.5	5	–	S	S	S	S	3	3	3	3	17	3	3.1	13.2
38	78	Post TURP	Membranous	3 × DVIU + TURP	BMG	25	24	26	24	22	–	MD	MD	MD	–	4	4	2	–	26	1	4.1	14.5
39	64	Post TURP	Penile and bulbar	3 × DVIU + TURP	BMG	36	37	36	35	35	–	S	S	S	–	5	5	5	–	25	19	2.5	11
40	71	Post prostate brachytherapy	Membranous	2 × DVIU	Cystostomy	22	20	20	20	–	–	S	S	S	–	3	3	3	–	18	35	0	0
41	74	Post TURBT and TURP	Penile	Meatotomy	Johansen	24	24	24	24	22	–	S	S	S	–	3	3	3	–	31	2	7	21
42	67	Idiopathic	Bulbar	10 × DVIU	BMG	28	29	28	23	25	–	S	S	S	–	5	5	3	–	22	2	4.5	14
43	70	Post TURP	Bulbar	TURP	End-to-end	14	24	22	23	22	–	S	S	S	–	3	3	3	–	32	4	4	21
44	48	Trauma	Bulbar	TURP	End-to-end	16	23	22	22.5	25	–	S	S	S	–	3	3	3	–	30	4	3.2	11
45	25	Trauma (straddle injury)	Bulbar	Newly diagnosed	BMG	60	72	71	71	70	–	MD	MD	MD	–	5	4	4	–	35	2	0	12
46	36	Trauma (PFUI)	Bulbar	1 × DVIU	End-to-end	4	6	4	5.5	5	–	M	M	M	–	3	2	2	–	33	6	0	8
47	58	Trauma (straddle injury)	Penile and bulbar + urethral fistula	3 × UP	BMG	11	15	64	64	60	–	MD	MD	MD	–	4	4	4	–	18	21	2.6	16
48	64	Post TURP	Bulbar	3 × DVIU	BMG	41	58	29	23	25	–	MD	MD	M	–	3	4	2	–	23	2	10	16.7
					r-BMG	6	3	4	6	3	–	S	S	S	–	4	3	3	–	32	4	7.7	18
49	45	Traumatic catheterisation	Bulbar	7 × DVIU	Johansen	20	11	20	22	15	–	MD	M	M	–	2	1	1	–	30	12	0	13.5
					r-end-to-end	9	6	7	8	3	2	MD	MD	MD	MD	3	4	4	2	16	4	7.5	17
50	53	Post URSL	Bulbar	20 × DVIU	BMG	23	22	25	21	20	–	MD	M	M	–	4	4	2	–	21	18	7	22
			Penile		r-BMG	26	25	35	30	30	–	S	MD	MD	–	5	5	5	–	8	3	9	24
51	70	Post TURP	Penile	2 × DVIU	BMG	23	26	23	22	20	–	S	S	S	–	3	3	3	–	1	1	8	11
			Bulbar		r-end-to-end	25	28	25	24	23	–	S	S	S	–	4	4	3	–		2	9	13
52	79	Post TURP	Bulbar	4 × DVIU	DVIU	5	8	5	5	4	–	S	S	S	–	3	3	3	–	14	12	8.4	9.1
			Bulbar and membranous		r-end-to-end	32	30	37	36	30	–	MD	MD	MD	–	5	5	5	–	23	20	6.4	11
53	64	Post TURP	Membranous	2 × DVIU + 2 × TURP	End-to-end	27	27	29	28	28	–	S	S	MD	–	5	5	5	–	32	3	6	12.5
			Bulbar		r-end-to-end	13	12	12.5	12	10	–	MD	MD	MD	–	2	2	2	–	33	6	9	11
54	46	Trauma (PFUI)	Penile and bulbar	2 × DVIU	End-to-end	12	12	10	10	12	–	S	S	S	–	3	3	3	–	32	11	0	9
			Penile		r-BMG	70	55	53	54	50	–	MD	MD	MD	–	4	4	4	–	8	20	0	11.5
55	68	TURP	Penile	2 × DVIU + TURP	BMG	2	6	5	6.5	7	–	S	S	S	–	3	3	3	–	8	21	0	11.5
					r-BMG	12	4	3	4	5	–	S	S	S	–	3	3	3	–	32	27	3	11

*PFUI* pelvic fracture urethral injury, *UP* uretropasty, *URSL* ureteroscopic lithotripsy, *TURP* transurethral resection of the prostate, *TURBT* transurethral removal of bladder tumour, *DVIU* direct visual internal urethrotomy, *RT* radiotherapy, *AMS* artificial urinary sphincter, *LS* Lichen sclerosus, *BMG* buccal mucosa graft, *r* reoperation, *NS* no stricture, *S* severe, *M* mild, *MD* moderate

## References

[1] Lumen N et al (2009) Etiology of urethral stricture disease in the 21st century. J Urol 182:983. https://www.ncbi.nlm.nih.gov/pubmed/19616805) 10.1016/j.juro.2009.05.023 19616805

[2] Cooperberg MR, McAninch JW, Alsikafi NF, Elliott SP (2007). Urethral reconstruction for traumatic posterior urethral disruption: outcomes of a 25-year experience. J Urol.

[3] Cunnigham JH (1910). The diagnosis of stricture of the urethra by Roentgen rays. Trans Am Assoc Genitourin Surg.

[4] McCallum RW, Colapinto V (1979). The role of urethrography in urethral disease. Part I. Accurate radiological localisation of the membranous urethra and distal sphincter in normal male subjects. J Urol.

[5] Jordan G, Chapple C, Heyns C (2012) An international consultation on urethral strictures. Société Internationale d’Urologie (SIU); 2012:201–233. ISBN: 978–0–9877465–2–8

[6] Rastogi R (2016). Can magnetic resonance urethrography (MRU) be a single-stop shop for male urethral stricture evaluation?. JOJ Urol Nephrol.

[7] Meeks JJ, Erickson BA, Granieri MA (2009). Stricture recurrence after urethroplasty: a systematic review. J Urol.

[8] McAnnich JW, Laing FC, Jeffrey RB (1988). Sonourethrography in the evaluation of urethral strictures: a preliminary report. J Urol.

[9] Krukowski J, Kałużny A, Kłącz J, Matuszewski M (2018). Comparison between cystourethrography and sonourethrography in preoperative diagnostic management of patients with anterior urethral strictures. Med Ultrason.

[10] Chiou RK, Anderson JC, Tran T (1996). Evaluation of urethral strictures and associated abnormalities using high resolution and color Doppler ultrasound. Urology.

[11] Sung DJ, Kim YH, Cho SB (2006). Obliterative urethral stricture: MR urethrography versus conventional retrograde urethrography with voiding cystourethrography. Radiology.

[12] Contrast Media Safety Committee ESUR (2018) Guidelines on Contrast Media v10. CMSC. http://www.esur-cm.org/index.php

[13] Dwivedi DK, Chatzinoff Y, Zhang Y, Yuan Q, Fulkerson M, Chopra R (2018). Development of a patient-specific tumor mold using magnetic resonance imaging and 3-dimensional printing technology for targeted tissue procurement and radiomics analysis of renal masses. Urology.

[14] Turner-Warwick R, Webster GD, Kirby R, King L (1993). Principles of urethral reconstruction. Reconstructive urology.

[15] Gupta N, Dubey D, Mandhani A, Srivastava A, Kapoor R, Kumar A (2006). Urethral stricture assessment: a prospective study evaluating urethral ultrasonography and conventional radiological studies. BJU Int.

[16] Landis JR, Koch GG (1977) The measurement of observer agreement for categorical data. Biometrics 33(1):159–174. 10.2307/2529310 ISSN 0006-341X JSTOR 2529310 (**PMID 843571**) 843571

[17] Baskin LS, Constantinescu SC, Howard PS (1993). Biochemical characterization and quantitation of the collagenous components of urethral stricture tissue. J Urol.

[18] Wood DN, Andrich DE, Greenwell TJ, Mundy AR (2006). Standing the test of time: the long-term results of urethroplasty. World J Urol.

[19] Nash PA, McAninch JW, Bruce JE (1995). Sono-urethrography in the evaluation of anterior urethral strictures. J Urol.

[20] Sheehan JL, Naringrekar HV, Misiura AK (2021). The pre-operative and post-operative imaging appearances of urethral strictures and surgical techniques. Abdom Radiol.

[21] Horiguchi A, Edo H, Soga S, Shinchi M, Masunaga A, Ito K, Asano T, Shinmoto H, Azuma R (2017). Pubo-urethral stump angle measured on preoperative MRI predicts urethroplasty type for pelvic fracture urethral injury repair. Urology.

[22] de Kemp V, de Graaf P, Fledderus JO, Ruud Bosch JLH, de Kort LMO (2015). Tissue engineering for human urethral reconstruction: systematic review of recent literature. PLoS ONE.

[23] Tao W, Bai G, Fu G, Niu X, Wang H, Wang G (2019). MR urethrography versus X-ray urethrography compared with operative findings for the evaluation of urethral strictures. Int Urol Nephrol.

